# Emerging Diagnostic Modalities in Oral Cancer Detection and Management

**DOI:** 10.31661/gmj.v13i.3423

**Published:** 2024-09-25

**Authors:** Ashkan Badkoobeh, Shadi Zarakhsh, Mehrnaz Fayazi, Pouya Kavianpour, Zahra Bahman, Maryam Onsori, Mahsa Etemadi

**Affiliations:** ^1^ Department of Oral and Maxillofacial Surgery, School of Dentistry, Qom University of Medical Sciences, Qom, Iran; ^2^ Faculty of Dentistry, Isfahan, Khorasgan Branch, Islamic Azad University, Isfahan, Iran; ^3^ School of Dentistry, Islamic Azad University of Medical sciences, Tehran, Iran; ^4^ Department of Endodontics, School of Dentistry, Ahvaz Jundishapur University of Medical Sciences, Ahvaz, Iran; ^5^ Faculty of Dentistry, Belarusian State Medical University, Belarus, Minsk; ^6^ Department of Operative Dentistry, Shahid Sadoughi University of Medical Sciences, Yazd, Iran; ^7^ Department of Periodontology, School of Dentistry, Tehran University of Medical Sciences, Tehran, Iran

**Keywords:** Oral Cancer, Diagnostics, Salivary Biomarkers, Optical Imaging, Liquid Biopsy, PET-CT, MRI Spectroscopy, Artificial Intelligence, Early Detection, Personalized Medicine

## Abstract

Oral cancer remains a significant global health concern, yet it is often detected at an advanced stage. The limitations of traditional diagnostic techniques have prompted increased research efforts towards the development of more efficient and early detection methods. Recent advancements in oral cancer diagnosis include the use of salivary biomarkers, optical imaging, liquid biopsy, advanced imaging, and AI algorithms. These non-invasive and painless sampling methods have shown high sensitivity and specificity, particularly in the case of salivary biomarkers. Clinical trials and cooperation are necessary to demonstrate the effectiveness of these technologies and gain approval from relevant authorities and acceptance among clinicians. This review highlights the potential of these new modalities in transforming the approach to oral cancer diagnosis, leading to early detection, accurate diagnosis, and quality treatment for patients, ultimately reducing the global burden of this disease.

## Introduction

Oral cancer remains a significant global health concern, accounting for approximately 3% of all cancer cases and ranking among the top ten most prevalent cancers worldwide [1][2]. Despite advances in therapeutic strategies, the prognosis for oral cancer patients often remains poor, primarily due to late-stage diagnosis and the inadequacies of traditional diagnostic methods [3]. Early detection is crucial for improving survival rates, as it enables earlier, less invasive treatment interventions [4]. The current standard diagnostic approach involves visual examination and histopathological analysis via biopsy, which, while essential, is associated with challenges such as invasiveness, subjective interpretation, and delayed results [5]. These limitations underscore the urgent need for more precise, advanced, and non-invasive diagnostic techniques [6].

Recent years have witnessed significant progress in the development of innovative diagnostic strategies for oral cancer. These emerging approaches utilize advancements in molecular biology, imaging technologies, and artificial intelligence to improve early detection and management. For example, salivary biomarkers provide a non-invasive method for detecting molecular changes specific to oral cancer [7]. Optical imaging techniques, such as fluorescence imaging and narrow band imaging (NBI), enable real-time, high-resolution visualization of oral tissues, which can facilitate the identification of malignancies at earlier stages [8]. Also, nanotechnology‑based detection and diagnostic methods, including nano‑based molecular imaging and nano‑based ultrasensitive biomarker detection, are gaining recognition as promising tools for early cancer detection and ongoing monitoring [9]. The integration of artificial intelligence (AI) in imaging analysis is revolutionizing the diagnostic landscape by enhancing accuracy and predictive capabilities [10]. Moreover, the development of point-of-care devices and portable diagnostic tools is increasing accessibility and efficiency, making advanced diagnostic capabilities available even in resource-limited settings [4].

This review aims to provide a comprehensive evaluation of these emerging diagnostic modalities in oral cancer detection, advantages, and limitations, and discussing their potential impact on clinical practice and future research directions

## Current Diagnostic Challenges

Conventional methods for assessing oral cancer, including clinical sign evaluation, visual inspection, palpation, tissue biopsy, and histopathological examination, have long been central to clinical practice [8]. While these techniques are essential, they present significant challenges. There is often considerable variability in outcomes depending on the clinician’s expertise, which may be inadequate for detecting early or subtle lesions, leading to delays in diagnosis and treatment [6][11]. Although biopsy remains the gold standard for accuracy, it is invasive, generally painful for the patient, and carries risks of complications such as infection and bleeding [12]. Also, histopathological analysis is time-consuming and can be influenced by inter-observer variability, further impacting the reliability of diagnose [4][6]. The heavy reliance on these conventional methods contributes to delayed diagnoses, with most cases of oral cancer being identified at advanced stages [8][13]. By the time treatment begins, survival rates are significantly reduced, a critical issue given the invasive and rapidly progressing nature of oral cancer [14]. Recent studies highlight the inadequacies of most current diagnostic methods, which stresses the importance of developing new diagnostic tools that are more sensitive, earlier, and cheaper [4][9]. Oral cancer screening is critical because when it is diagnosed at an early stage, treatment is proportionally more effective, and mortality is much higher [10]. For example, the 5-year survival rate is 88% for stage I and 50.9% for stage IV [15]. Also, Marzouki et al. [16] found that detecting oral cancer at an early stage is strongly correlated with improved disease-free survival, indicating that patients diagnosed in the early stages of the disease have a significantly higher likelihood of remaining free from cancer following treatment.

Furthermore, while the 5-year survival rate for oral cancer patients has remained relatively unchanged over the years, advanced diagnostic methods show significant potential for enhancing early and precise detection and treatment [17]. This underscores the importance of improving diagnostic accuracy and developing tools to identify oral cancer in its early stages as key priorities in managing this disease [18].

As addressed above, these challenges can be solved by the use of emerging diagnostic technologies. Techniques involving molecular biology have been used to find that biomarkers in the saliva can identify molecular changes linked to the disease at an early stage [17][19][20]. They are non-invasive biomarkers, impose no cost to the healthcare system, and can be obtained readily from patients, especially in health facilities where there are very limited available diagnostic services [20][21]. Fluorescence imaging and narrow-band imaging enhance real-time visual features of tissue changes, making the diagnosis of precancerous and malignant lesions easier [22][23].

Targeted molecular markers, non-invasive liquid biopsy, and other genomic and epigenomic approaches may prove very effective in diagnosing oral cancer and following up with patients [9][17]. Circulating tumor DNA or other biomarkers in blood or other fluids is amenable to repeated sampling as it is the least invasive procedure for diagnosing and tracking diseases, in addition to monitoring response to treatment [10].

Also, the incorporation of AI in the analysis of diagnostic images leads to increased precision and decreased intra-observer variation, as it offers accurate objective measures [24][25].

There is also an economic aspect to consider when it comes to the creation of new diagnostic technologies. Proposed technologies must be cost-effective to reach all the intended consumers [4].

It seems these technologies can help bridge the gap in cancer care, enabling early detection and timely intervention for a broader population [4][10].

## Emerging Diagnostic Modalities

Currently, there are revolutionary changes in the diagnosis of oral cancer with new technologies and methodologies. These new beginning diagnostic techniques have the potential to better earlier diagnosis, deliver more accurate results, and more economically friendly results in terms of outcomes in patients and health systems. Down below we describe several innovative strategies that are cutting-edge approaches for the diagnosis of oral cancer. In Table-1, we compare strengths and limitations across various factors of these methods.

Salivary Biomarkers

Thus, comparing it with other methods for diagnosing oral cancer, salivary diagnostics has proven itself to be non-invasive and easily accessible for the patient [7][20]. Saliva also consists of nucleic acids, proteins, enzymes, and other small molecules which ideally offer the salivary biomarkers for early diagnosis of oral cancer [37][38]. Chronic inflammation of the oral cavity and consumption of tobacco and alcohol products lead to molecular changes in saliva samples that can be analyzed to detect signs of oral cancer at its early stage along with disease progression [7][39][40]. Research has pointed out numerous messages, miRNA, and proteins that are being discussed in the range of potential salivary biomarkers capable of discriminating neoplastic tissues from healthy ones [20][41].

## Optical Imaging Techniques

**Table:** Table1. Emerging Diagnostic Modalities for Oral Cancer, Highlighting Their Strengths and Limitations Across Various Factors.

**Modality**	**Sensitivity**	**Specificity**	**Cost**	**Accessibility**	**Patient Comfort**	**Compliance**
**Salivary Biomarkers [7] **	85% - 90%	80% - 85%	Low to moderate	High (non-invasive, portable)	High (non-invasive, no pain)	High
**Fluorescence Imaging [8,11] **	80% - 90%	70% - 85%	Moderate	Moderate (requires special equipment)	Moderate (non-invasive)	Moderate
**Narrow Band Imaging (NBI) [26–28] **	93% - 95%	80% - 90%	Moderate to high	Moderate (requires endoscopic equipment)	Moderate (non-invasive)	Moderate
**Confocal Laser Endomicroscopy [29] **	86.8%	92%	High	Low to moderate (specialized equipment)	Low to moderate (invasive, localized discomfort)	Low to moderate
**Liquid Biopsy [30]in accordance with best evidence practice. Liquid biopsy(LB) **	85% - 90%	85% - 90%	Moderate to high	High (non-invasive, accessible)	High (non-invasive, minimal discomfort)	High
**Genetic and Epigenetic Markers [7,31] **	85% - 95%	80% - 90%	High	Moderate (lab-based, requires specialized personnel)	Moderate (requires sample collection)	Moderate
**Positron Emission Tomography (PET) [32–34] **	96%-98%	80% -93%	Very high	Low (specialized equipment, high cost)	Low (involves radiation exposure, long procedure)	Low
**Magnetic Resonance Imaging (MRI) [35] **	76.4%	91.3%	High	Moderate (requires specialized equipment)	Moderate to low (non-invasive but lengthy)	Moderate
**AI in Imaging Analysis [18] **	97.76% - 99.26%	92% - 99.42%	Moderate to high	Moderate (depends on integration with imaging equipment)	High (enhances interpretation accuracy)	High
**Point-of-Care Devices [4,36]as compared to the gold standard test (histopathology) **	86.8% - 92%	83.6% - 94.5%	Low to moderate	High (portable, easy to use)	High (non-invasive, user-friendly)	High

Optical imaging technologies are leading to a significant improvement in the visualization of tissues inside our mouths, and as for now, one can get a picture of his or her mouth showing early signs of disease [26][42][43].

• Fluorescence Imaging: This technique involves the use of dyes that lodge in cancer tissue, and when exposed to certain light intensity, they come to identifiable coloration. Imaging using fluorescence has been discovered to have better features as a technique to improve visualization of lesions in the mouth that could hardly be seen through regular imaging [11][42][44].

• Narrow Band Imaging (NBI): NBI enables favorable features of blood vessels and the mucosa, due to the selective use of light wavelengths. This technique enhances the visualization of vascular changes that are often linked to initial neoplastic events in mucosal tissues of the oral cavity [27][28].

• Confocal Laser Endomicroscopy: Because it is a hybrid imaging mode, OCT can offer cross-sectional images of oral tissues at the microscopic level in real time and without the need for excisional biopsy. It enables physicians to detect tissue dysfunctions, including cellular transformations and structures, to diagnose diseases at early stages without the help of biopsy [29][45].

Molecular Techniques

Molecular approaches have revealed an increasing interest in the detection of oral cancer due to their increased sensitivity and specificity [20][46].

Liquid Biopsy: This is a noninvasive diagnostic approach that can rely on various biomarkers such as circulating tumor DNA (ctDNA), RNA, and others from biofluids including blood or saliva samples [30][46]. This includes technology to perform liquid biopsy that provides genetic and epigenetic information on markers of cancers, particularly, oral cancer that can be used for diagnosis, monitoring of treatment, and prognosis [12][39][46].

• Genetic and Epigenetic Markers: The present literature study has revealed that the prospects in genomics and epigenomics have provided the molecular and genetic foundations behind the causation of oral cancer [31]. These markers can be PCR, NGS, and methylation assays. Exploring such markers is proving to be more precise and comprehensive for cancer diagnostics [7][46].

## Advanced Imaging Technologies

• Positron Emission Tomography (PET): PET scan relies on the usage of radioactive isotopes to identify the amount of metabolic activity in tissues. In oral cancer, PET has proven useful in identifying areas exhibiting a high metabolic rate, indicating malignancy, which helps in the identification of both primary and metastatic tumors [32][33][34].

• Magnetic Resonance Imaging (MRI): MRI ensures visualization of the details of the anatomy of the oral structures and structures around them. Functional MRI and diffusion-weighted imaging can give additional information on the tumor microenvironment and detect early anatomical alterations that may translate to cancer [13][35].

• AI in Imaging Analysis: Machine learning techniques are becoming the perfect tools to augment imaging platforms for improved diagnosing capabilities. Some studies showed AI can quantify values and perform complex computations with large sets of data, figure out the potential patterns, and generate analytical reports without intervention and interferences, which is inevitable in traditional image interpretation [18][24][25].

## Point-of-Care Devices

Synergistically, portable diagnostic technologies and point-of-care devices are now driving more services and more sophisticated technologies into the emergent markets, particularly in the context of resource-limited settings [4][43]. These gadgets that are fashioned to be portable, affordable, and efficient can generate results within minutes. Some of them include handheld optical imaging devices, portable intensified PCR machines, and diagnostic tools based on saliva sampling. These technologies aid in the identification and surveillance of oral cancer before patients can even go for medical attention with their woes [21][36].

## Clinical Application

It is however important to remember that new diagnostic technologies have to be confirmed or ruled out for use in clinical practice from clinical trials.

Several research and clinical assessment exercises have been carried out to determine the effectiveness, specificity, and usability of the new technologies in the early identification and treatment of oral cancer.

in a systematic review, Khijmatgar et al. [47] identified that several salivary biomarker signature such as chemerin, MMP-9, Phytosphingosine, Pipecolinic acid have about 80% sensitivity and 90% specificity in detection of oral squamous cell carcinoma.

Liquid biopsy techniques have been considered in some contexts. Olms et al. [48] Liquid-based cytology compere conventional cytology is simplifying cell collection and there are less transfer mistakes.

Some studies on fluorescence imaging and NBI have been performed to determine the efficacy of optical imaging techniques. For instance, an investigation by Takano et al. [49] showed that NBI increased the chances of visualizing the vascular pattern linked to early neoplastic transformation of mucosal tissues of the oral cavity. These features implied improved clinical results since the probability of early cancer detection and subsequent treatments was escalated [26][49].

## Future Directions

Oral cancer diagnosis is currently an exciting area of development that is steadily seeing the incorporation of more new technologies and advancements [50]. the integration of salivary biomarkers, optical imaging, liquid biopsy, and advanced imaging technologies makes it a single integrated system for diagnosis [9][7]. This is a very effective approach as it covers multiple aspects of the patient’s health and assists in the diagnostic process and development of further treatment plans [49][51].

Another significant innovation that can redefine oral cancer diagnosis is the point-of-care and portable diagnostic equipment. These devices are easy to use, comparatively inexpensive, and can be especially helpful in areas with limited resources where correct and swift diagnostics are needed [52]. Furthermore, the use of AI and machine learning in diagnostic imaging also holds the prospect of improvement of diagnostic capacities [53]. Predictive analytics could provide real-time risk analysis and prognosis data; Integration of AI into the telemedicine framework could enable remote diagnostic capabilities and make expert care more accessible [54].

To support these innovative diagnostic technologies to become a standard of care, further clinical acceptance and satisfactory regulatory review must be established. Clinical trials and continued research on such treatments are important in proving the effectiveness and safety of these techniques. This will require close cooperation among the researchers, clinicians, and the regulating authorities since the treatment and the trials will have to conform to set standards and guidelines.

## Conclusion

Currently, there is great progress in technology and molecular biology that significantly changes the methods of oral cancer diagnostics. Therefore, novel diagnostic methods such as salivary biomarkers, optical imaging, liquid biopsies, enhanced imaging, and predictive analytics by AI present groundbreaking opportunities. There is a possibility of sampling saliva from patients since the biomarkers can be measured from saliva samples, a non-invasive and easily accessible method; several previous studies have shown high sensitivity and specificity. Fluorescence imaging and narrow band imaging in addition to other optical imaging techniques again boost the process of visualization of early neoplastic changes besides improving the detection rates. Through liquid biopsy, it is possible to find circulating tumor DNA and other molecules, which makes it possible to have a minimally invasive approach for disease progression and therapeutic intervention assessment.

Enduring diagnostic imaging technologies like PET-CT and MRI spectroscopy provide clearer anatomical and metabolic image information and improve diagnostic certainty and accurate tumor delineation and staging. Recent studies have shown enhancing the utilization of AI in imaging analysis increases diagnostic accuracy and decreases the variability to reach a higher level. point-of-care testing, which involve the use of ‘democratic’ diagnostic equipment, means that advanced testing is less out of reach for resource-scarce environments underlining the future possibilities for utilization and therefore the health impact.

Oral cancer screening methods still require significant improvements, and further research is essential to explore and apply these novel techniques in this field. This includes investigating new biomarkers, developing advanced imaging technologies, and integrating AI to enhance the accuracy and efficiency of early detection and diagnosis. The continued advancement and refinement of these innovative approaches will be crucial in improving patient outcomes and reducing the overall burden of oral cancer.

## Conflict of Interest

The authors declare no conflict of interest.
